# Enhanced activity of apramycin and apramycin-based combinations against *Mycobacteroides abscessus*

**DOI:** 10.1093/jac/dkaf433

**Published:** 2025-11-28

**Authors:** Yanqin Huang, Katherine A Truelson, Isabella A Stewart, George A O'Doherty, James E Kirby

**Affiliations:** Department of Pathology, Beth Israel Deaconess Medical Center, Boston, MA, USA; Harvard Medical School, Boston, MA, USA; Department of Pathology, Beth Israel Deaconess Medical Center, Boston, MA, USA; Department of Pathology, Beth Israel Deaconess Medical Center, Boston, MA, USA; Department of Chemistry, Northeastern University, Boston, MA 02115, USA; Department of Pathology, Beth Israel Deaconess Medical Center, Boston, MA, USA; Harvard Medical School, Boston, MA, USA

## Abstract

**Background:**

*Mycobacteroides abscessus* is a rapidly growing non-tuberculous mycobacterium that causes chronic lung and soft tissue infections. Treatment options are severely limited. Amikacin has historically been a mainstay of combination treatment regimens. However, irreversible hearing loss and vestibular toxicity have led to a search for alternative agents. Apramycin is a novel aminoglycoside currently in Phase I clinical trials that may offer lower potential for ototoxic and renal toxic side effects.

**Objectives:**

The goal of this study was to compare apramycin's *in vitro* activity with amikacin and other aminoglycosides against a large collection of *M. abscessus* clinical isolates, alone, and for apramycin and amikacin, in combination with clofazimine or linezolid. We also examined activity against a more limited collection of other rapidly growing mycobacteria.

**Methods:**

Analysis was performed using reference broth microdilution MIC testing, inkjet printer-assisted checkerboard assays, and time–kill assays.

**Results:**

Against *M. abscessus*, the MIC_50/90_ for apramycin (2 mg/L) was one-eighth that of amikacin (16 mg/L). Plazomicin was inactive, and organisms were rarely susceptible to tobramycin. Synergy of either apramycin or amikacin with clofazimine or linezolid was not detected by checkerboard assay. In time–kill studies, clofazimine modestly increased activity of apramycin and, to a lesser extent, amikacin. Apramycin and amikacin showed delayed bacterial killing that either achieved or approached a bactericidal threshold. Apramycin was similarly potent against other rapidly growing mycobacteria tested.

**Conclusions:**

Apramycin exhibits more potent *in vitro* activity against a diverse set of *M. abscessus* and other rapidly growing mycobacteria than currently recommended aminoglycosides.

## Introduction

*Mycobacteroides abscessus* is a rapidly growing mycobacterium that causes chronic progressive lung and soft tissue infections.^1^ It is often MDR, and prolonged treatment courses are required for suppression, control or cure when possible, leading to cumulative toxicities. The cure rate for lung infection is ∼30%–50% for *M. abscessus* ssp. *abscessus,* with surgical intervention often needed for infection site control.^1^

Currently combinatorial therapy includes a choice of two to five antimicrobials guided by antimicrobial susceptibility testing. Amikacin is one of the recommended parenteral agents for initial combination therapy in updated American Thoracic Society/European Respiratory Society/ESCMID/IDSA Clinical Practice Guideline guidelines for *M. abscessus* lung infection.^2,3^ However, there is no consensus on length of amikacin treatment; in practice, IV agents such as amikacin are often used for a month or longer.^2^

Unfortunately, the toxicities of extended aminoglycoside treatment include irreversible vestibular and cochlear damage, and renal toxicity. Apramycin is a novel aminoglycoside with a mono-substituted 2-deoxystreptamine attached to a bicycle sugar containing disaccharide having a shifted binding site in the 30S ribosome compared with amikacin. In a human Phase I clinical trial (NCT04105205) apramycin was safe (results not published); and in the rat model, renal toxicity appeared to be much lower than for gentamicin.^4^ In an explanted cochlear model, apramycin did not appear to be associated with ototoxicity, potentially because of lower affinity for mitochondrial and/or eukaryotic cytoplasmic ribosomes.^4–8^ It is now being studied in a second Phase I trial in the USA (NCT05590728) to determine lung epithelial lining fluid and serum pharmacokinetics.

An early disc diffusion study found that apramycin was active *in vitro* against *M. abscessus* clinical isolates; however, quantitative MIC data were not assessed.^9^ A more recent study found apramycin MIC values of 0.5 mg/L for six *M. abscessus* clinical isolates: two isolates each of ssp. *abscessus*, *bolletii* and *massiliense*.^10^ In this limited analysis, apramycin was found to have lower MICs than amikacin. In contrast to amikacin, apramycin was also found in time–kill study analysis to be bactericidal for the four *M. abscessus* isolates examined. Genetic experiments support the role of the multi-acetyltransferase, Eis2, which is ubiquitously present in *M. abscessus*, as the direct cause of reduced amikacin activity relative to apramycin, as only amikacin is a substrate for this resistance enzyme.^10–12^ In addition, apramycin activity unlike that of amikacin was not affected by the transcriptional regulator, WhiB7, which up-regulates a set of genes conferring resistance to several frontline antimicrobial therapies, in the case of amikacin through up-regulation of Eis2.^13^

*In vivo* experimental evidence suggests that apramycin is a promising treatment for lung and soft tissue infections, the major sites of *M. abscessus* disease.^10,14^ Murine studies showed that apramycin lung epithelial lining fluid levels approximate those in plasma. In turn, apramycin proved efficacious in treatment of murine pneumonia models of infection caused by *Klebsiella pneumoniae*, *Acinetobacter baumannii*, *Pseudomonas aeruginosa* and *M. abscessus.* Apramycin was also shown to be active in thigh and urinary tract models.^4,8^ Pharmacokinetic/pharmacodynamic (PK/PD) modelling indicates a high likelihood of apramycin target attainment in lung infection with the previously mentioned Gram-negative pathogens in humans dosed at 30 mg/kg and a pathogen MIC ≤8 mg/L,^14^ providing a potential categorical breakpoint for *M. abscessus* susceptibility.

Taken together, existing data suggest that apramycin might have more potent activity and lower toxicity compared with amikacin for *M. abscessus* infection. This current work significantly extends activity spectrum analysis of apramycin in comparison with other aminoglycosides against contemporary clinical isolates of *M. abscessus* and other rapidly growing mycobacterium species, and tests apramycin's potential *in vitro* synergistic activity in combination with other antimicrobials used for *M. abscessus* treatment.

## Materials and methods

### Antibiotics

Apramycin sulphate (Alfa Aesar, MA, USA, Cat# J66616, lot#Y05F120); clofazimine (Acros Organics, Geel, Belgium, Cat# 461760010, lot# A0395020); linezolid (Acros Organics, cat# 460592500, lot# A0400349); tobramycin sulphate (Research Products International, IL, USA, cat#T45000-1.0, lot#10298-155238); plazomicin sulphate (ToKu-E, WA, USA, cat #P140, lot#P140-01US); and amikacin disulphate (Alfa Aesar, cat#J63862.14, lot# N08H025) were obtained from indicated sources. Aminoglycosides (sulphate salts) were dissolved according to CLSI guidelines using lot-specific activity values provided by the manufacturer to correct for potency.^15^

### Bacterial isolates

Clinical isolates were from Beth Israel Deaconess Medical Center (Boston, MA, USA) from years 2016–2023 under an Institutional Review Board–approved protocol and from the ATCC (Manassas, VA) as listed in Table S1 (available as Supplementary data at *JAC* Online). All isolates and strains were stored at −80°C and minimally passaged until use. Subspeciation was determined by a previously described multiplex PCR typing method^16^ and/or by the University of Tyler, TX, Mycobacteriology and Nocardia Reference Laboratory.

### MIC testing

Broth microdilution antimicrobial susceptibility testing was performed as recommended by the CLSI.^17^ Serial 2-fold dilutions of freshly prepared drugs were dispensed using the HP D300 digital dispensing system (HP, Inc., Palo Alto, CA, USA) into sterile, round-bottom, polystyrene 96-well plates (Evergreen Scientific, Los Angeles, CA, USA) to achieve final desired antimicrobial concentrations as we previously described.^18–22^  *Mycobacterium peregrinum* ATCC 700686 and *Escherichia coli* ATCC 25922 were used as quality control strains.^23^ Following incubation at 30°C in ambient air, MIC results were recorded from Day 3 to Day 5 per CLSI recommendations.

### Checkerboard synergy assays

Antimicrobial combinations of apramycin or amikacin with linezolid and clofazimine were dispensed using the HP D300 digital dispensing system (HP, Inc., Palo Alto, CA, USA) as previously described in full checkerboard synergy arrays^18,24^ and tested against three *M. abscessus* clinical isolates, NTM18, NTM27 and NTM28, in duplicate. The fractional inhibitory concentration index (FICI) was determined by summing the individual FICs for each antibiotic in each inhibited well where the FIC for each antimicrobial equals the inhibitory concentration of each antimicrobial in the combination divided by the MIC of the antimicrobial when tested alone. The FICI is the lowest summation for which complete visual growth inhibition was observed. The highest (most conservative) FICI among replicates was scored as indicating synergy (≤0.5), indifference (0.5 < FICI ≤ 4.0) or antagonism (FICI > 4.0), respectively.^25^

### Time–kill assays

Time–kills assays were performed in duplicate, as previously described, against NTM27 and NTM28 with a starting inoculum of ∼10^6^ cfu/mL.^2^ Apramycin or amikacin was tested either alone or combination at indicated multiples of MICs determined by broth microdilution. Bacteria were incubated at 30°C with sampling for cfu determination at indicated timepoints. Bactericidal activity was defined as a ≥3 log_10_ reduction in viable counts relative to the initial inoculum after the incubation period indicated. Synergy was defined as a ≥2 log_10_ reduction in viable counts at 72 h in the antimicrobial combination treatment group relative to the most active single agent tested alone at the same concentrations.

### Spontaneous resistance frequency

Log-phase NTM27 and NTM28 *M. abscessus* ssp. *abscessus* were plated at 10^9^, 10^10^ or 10^11^ cfu onto cation-adjusted Mueller–Hinton agar plates containing apramycin or amikacin at a concentration 4-fold or 8-fold greater than the antibiotic specific MIC. The resistance frequency was calculated by determining the fraction of bacteria growing on antibiotic-containing versus non–antibiotic-containing medium after 5 days of incubation at 30°C.^26^

## Results

### MIC data

The MIC_50,_ MIC_90_ and MIC ranges for antimicrobials tested are listed in Table 1. The *M. abscessus* isolate subspecies, specimen source and individual MIC values for each isolate are listed in Table S1. Notably, the MIC_50_ and MIC_90_ values for apramycin were one-eighth that of amikacin. Plazomicin was inactive at the highest concentration tested, whereas only 17.5% of the isolates were predicted to be susceptible to tobramycin. A smaller number of *Mycobacterium fortuitum* and *Mycobacterium chelonae* were also tested with similar apramycin activity and improved amikacin activity compared with *M. abscessus* (Table S2).

**Table 1.** MIC_50_, MIC_90_ and MIC range in mg/L for aminoglycosides and antimicrobials used in synergy testing

Antimicrobial	*n* ^a^	MIC_50_^b^	MIC_90_^b^	MIC range	%S^c^
Apramycin	56	2	2	1–16	96.5
Amikacin	56	16	16	1–32	91.2
Plazomicin	39	>16	>16	>16	
Tobramycin	39	8	16	1–32	12.8
Clofazimine	17	0.25	4	0.06–8	
Linezolid	17	8	32	1–32	64.7

^a^Number of isolates tested.

^b^Values shown were determined from the modal MIC of three biological replicates per isolate.

^c^Percent susceptible (S) calculated where CLSI M62 breakpoints are available^23^ with the exception of apramycin susceptibility breakpoint of ≤8 mg/L, which is based on predicted target attainment with a 30 mg/kg humanized dose as described elsewhere.^27^ CLSI amikacin susceptibility breakpoint for *M. abscessus* is currently ≤16 mg/L.

### Checkerboard synergy testing

FICI values for combinations of apramycin or amikacin with clofazimine and linezolid against three representative clinical isolates are shown in Table 2. Clofazimine and linezolid were chosen as partner antibiotics for these studies based on compelling activity spectra from our prior studies and oral dosing options for these drugs, potentially simplifying outpatient treatment regimens.^22^ However, indifference was observed for all combinations against all isolates.

**Table 2.** FICI values for checkerboard testing of apramycin or amikacin in combination with clofazimine or linezolid^a^

	Clofazimine	Linezolid
**Apramycin**	2, 1.5, 1.75	1.5, 1.5, 1.5
**Amikacin**	2, 3, 2.5	1.5, 3, 2

^a^FICI values, separated by commas, are listed separately for *M. abscessus* ssp. *abscessus* clinical isolates NTM18, NTM27 and NTM28, respectively.

### Time–kill studies

**Figure 1.**
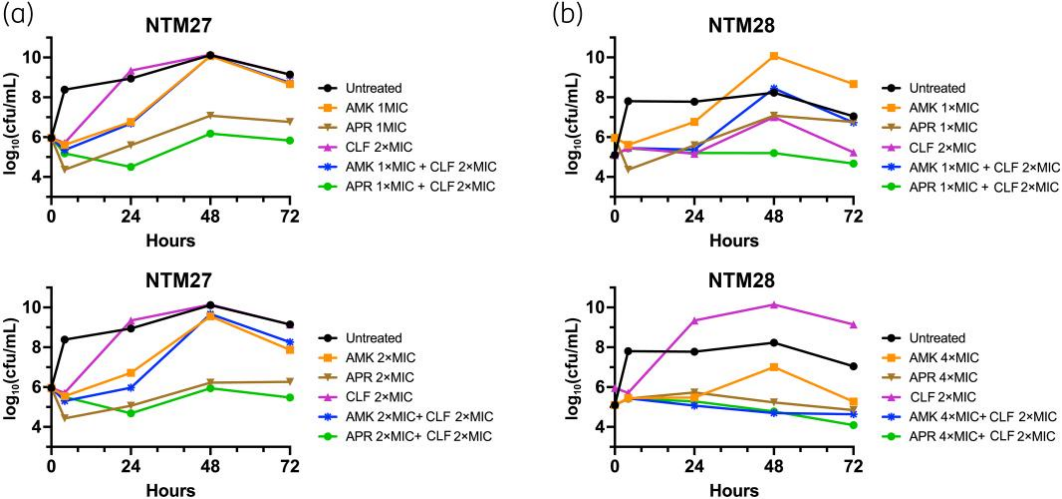
Time–kill analysis of amikacin and apramycin alone and in combination with clofazimine against two *M. abscessus* ssp. *abscessus* clinical isolates. (a) Time–kill analysis against strain NTM27 using concentrations of antimicrobials at indicated multiples of MIC values determined by the reference broth microdilution method. (b) Time–kill analysis for NTM28. For NTM27, MICs for AMK, APR and CLF were 8, 2 and 0.25 mg/L, respectively. For NTM28, MICs for AMK, APR and CLF were 8, 2 and 4 mg/L, respectively. AMK, amikacin; APR, apramycin; CLF, clofazimine.

**Figure 2.**
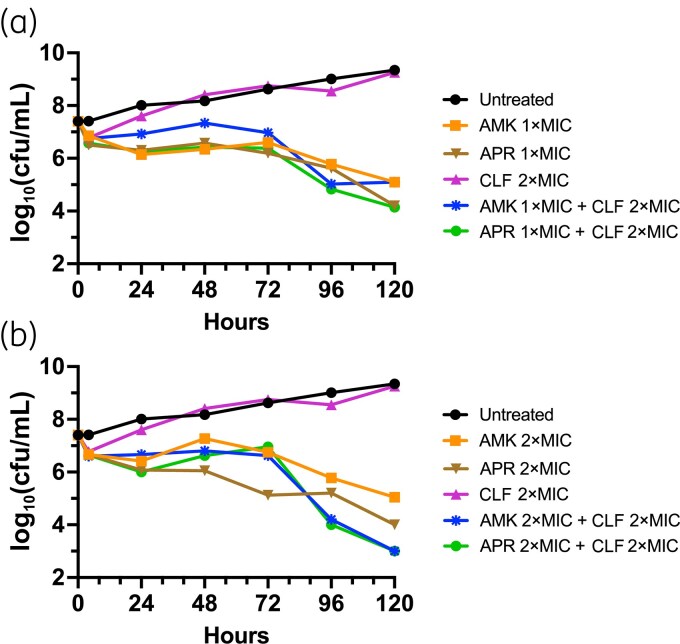
Extended incubation reveals slow bactericidal killing by apramycin and amikacin, modestly potentiated by clofazimine. Time–kill analysis during extended incubation was performed for NTM27 *M. abscessus* ssp. *abscessus* with antibiotic concentrations at indicated multiples of MIC values determined by reference broth microdilution. (a) Amikacin and apramycin added at 1×MIC alone or in combination with clofazimine. (b) Amikacin and apramycin added at 2×MIC alone or in combination with clofazimine. Abbreviations: AMK, amikacin; APR, apramycin; CLF, clofazimine.

Time–kill analysis for comparison of effects of apramycin and amikacin alone and in combination with clofazimine was performed against two representative *M. abscessus* ssp. *abscessus* clinical isolates (Figure 1). Apramycin, but not amikacin, at 2×MIC reduced cfu at 72 h by >3 log_10_ for NTM27, but not for NTM28, compared with untreated controls. Clofazimine alone was not fully bacteriostatic or without effect at 2× its MIC. However, at this concentration, it appeared to modestly potentiate the activity of apramycin, as a ≥3 log₁₀ reduction in cfu compared with untreated controls was now also observed at 1×MIC for NTM27 and at 4×MIC for NTM28. However, neither aminoglycoside was synergistic with clofazimine at 2×MIC, based on the standard threshold criterion of a ≥2 log_10_ decrease in cfu/mL in combination testing compared with the most active single agent. In experiments with extended 5 day incubations, both apramycin and amikacin at 1× and 2×MIC demonstrated continued, slow killing of NTM27, with apramycin achieving bactericidal activity and amikacin approaching this threshold (Figure 2). The combination of either apramycin or amikacin at 2×MIC with clofazimine at 2×MIC was bactericidal, achieving a ≥4 log₁₀ kill by Day 5, effectively sterilizing cultures to the limit of detection for NTM27. NTM28 was not tested in extended time–kill experiments.

### Spontaneous resistance mutation frequency

Both apramycin and amikacin at 4×MIC and 8×MIC showed a low and essentially identical spontaneous resistance frequency against two *M. abscessus* ssp. *abscessus* clinical isolates (Table 3).

**Table 3.** Spontaneous resistance frequency in representative *M. abscessus* ssp. *abscessus* isolates at indicated multiples of amikacin and apramycin MIC

Strain	Amikacin (MIC, 8 mg/L)		Apramycin (MIC, 2 mg/L)	
	4×MIC	8×MIC	4×MIC	8×MIC
NTM27	4 × 10^−9^	2 × 10^−9^	2 × 10^−9^	2 × 10^−9^
NTM28	4 × 10^−9^	7 × 10^−10^	4 × 10^−9^	3 × 10^−10^

## Discussion

Amikacin has long been an integral part of treatment regimens for *M. abscessus* infection. This general bactericidal property of aminoglycosides is thought to be advantageous in the rapid clearance of organisms, especially at sites where the efficacy of cell-mediated immunity may be limited.

However, there is concern that current breakpoints for amikacin may be set too high for lung infections. Notably, the amikacin susceptibility breakpoint for Enterobacterales was recently lowered by CLSI to ≤4 mg/L, reflecting current understanding of PK/PD relationships,^15^ and is significantly lower than the 16 mg/L susceptibility breakpoint for *M. abscessus*.^10,28,29^ Furthermore, results from hollow-fibre infection models suggest that dosing regimens required for reliable efficacy would invariably be associated with ototoxicity.^28^ In the present study, we found that the MIC_50_/MIC_90_ of amikacin across a large set of clinical isolates was 16 mg/L, suggesting that amikacin should offer limited clinical benefit for the direct treatment of pulmonary airway disease. Yet, there is still evidence of clinical efficacy in uncontrolled retrospective studies comparing different multidrug regimens with or without amikacin, despite PK/PD parameters that would typically suggest limited utility.^30,31^ Overall, however, amikacin appears far from an ideal choice in regimens where bactericidal activity is desired.

Therefore, a more potent, bactericidal antibiotic without the limiting side effects of amikacin would be welcome. Here, we found that apramycin was 8-fold more active (MIC_50_/MIC_90_) than amikacin by weight. The equivalence of MIC_50_ and MIC_90_ at a relatively high 16 mg/L for amikacin and low 2 mg/L for apramycin is indicative of a generally consistent activity of these two drugs for *M. abscessus* with rare outliers, which has implications for their use in current and future empirical treatment regimens. Recently, in a murine lung infection model, a human-equivalent apramycin dose of 30 mg/kg was found to offer a 99% probability of achieving a 2 log₁₀ reduction of *A. baumannii* for isolates with an MIC ≤16 mg/L.^32^ This MIC threshold, if found to be similarly applicable to *M. abscessus* infection, would encompass 100% of the largest reported set of *M. abscessus* clinical isolates examined to date.

In contrast to apramycin, tobramycin was generally inactive, with MICs roughly correlating with amikacin MIC values, whereas plazomicin was universally inactive. The *M. abscessus* genome encodes an AAC(2′) enzyme sharing 35% identity and 53% similarity with the aminoglycoside *N*-2′-acetyltransferase-Ia [AAC(2′)-Ia] from *Providencia stuartii*, which is known to inactivate plazomicin and tobramycin through acetylation of the *N*-2′ position of their 4-*O*-linked sugars.^12^ The alpha-fold–predicted structure of the *M. abscessus* protein is highly similar to the *Providencia* enzyme (PDB 6VRO) with a compelling root mean squared deviation of 0.8 Å (Figure S1). Therefore, this enzyme is a prime candidate for inactivation of both plazomicin and tobramycin in *M. abscessus*. However, we have not confirmed this hypothesis through genetic knockout experiments as this was not a specific focus of the current work. Nevertheless, the existence of a pre-existing resistance enzyme suggests that neither plazomicin nor tobramycin should be used for treatment of *M. abscessus* even in the infrequent cases that clinical laboratory testing indicates susceptibility for the latter.

The lack of formal synergy between apramycin or amikacin and clofazimine or linezolid examined in checkerboard testing (also observed in time–kill studies for the combination of aminoglycosides and clofazimine) suggests that subtherapeutic concentrations of either aminoglycoside (below those suggested by PK/PD relationships to be efficacious as single agents) should not be relied upon to provide benefit in combination treatment, which is a significant drawback for amikacin due to its much higher MIC levels. However, the results also suggest some enhanced killing with clofazimine/apramycin combinations, which may translate to improved therapeutic efficacy.

A similar rate of spontaneous resistance to apramycin and amikacin, consistent with an overlapping, 16S rRNA target (Figure S2), suggests that resistance to apramycin is unlikely to develop more rapidly than amikacin during treatment. However, a limitation of our study is that we did not define the repertoire of spontaneous 16S rRNA resistance mutations, which may be more or less likely to arise *in vivo* based on their effect on bacterial fitness. We expect that the infrequently identified, but most frequently described mutation in clinical *M. abscessus* isolates responsible for constitutive high-level resistance to amikacin, 16S rRNA A1408G,^33^ would confer similar high-level resistance to apramycin. This prediction is based on our observations of the 16S rRNA A1408G mutation conferring high-level resistance to apramycin in single *rrn* operon *E. coli*.^34^ Therefore, for both drugs, the presence of a single-copy *M. abscessus* rRNA operon highlights the additional necessity of combination treatment to prevent spontaneous, single-step resistance leading to treatment failure.^35^

In conclusion, apramycin shows significant promise as a potent, bactericidal agent against the vast majority of *M. abscessus* isolates. Our findings support further investigation of apramycin for the treatment of *M. abscessus* infections either by IV or nebulized administration routes.

## Supplementary Material

dkaf433_Supplementary_Data

## Supplementary data

Figures S1 and S2 and Tables S1 and S2 are available as Supplementary data at *JAC* Online.
