# Supplementary material for: Enhanced activity of apramycin and apramycin-based combinations against Mycobacteroides abscessus
Source: J Antimicrob Chemother. 2025 Nov 28;81(1):dkaf433. doi: 10.1093/jac/dkaf433 (PMC12802941; doi:10.1093/jac/dkaf433)
Supplement: dkaf433_Supplementary_Data [file dkaf433_supplementary_data.docx]

**SUPPLEMENTARY FIGURES AND TABLES**

**Enhanced Activity of Apramycin and Apramycin-Based Combinations Against *Mycobacteroides abscessus.***

Yanqin Huang^a,b^, Katherine A. Truelson^a^, Isabella A. Stewart^a^, George A. O'Doherty^c^, James E. Kirby^a,b,#^

a

^a^Department of Pathology, Beth Israel Deaconess Medical Center, Boston, MA, USA

^b^Harvard Medical School, Boston, MA, USA

^c^Department of Chemistry, Northeastern University, Boston, MA 02115, USA

Running Head: Apramycin combinations against *M. abscessus*

^#^Address correspondence to: James E. Kirby, jekirby@bidmc.harvard.edu

**Supplementary Figures**

**
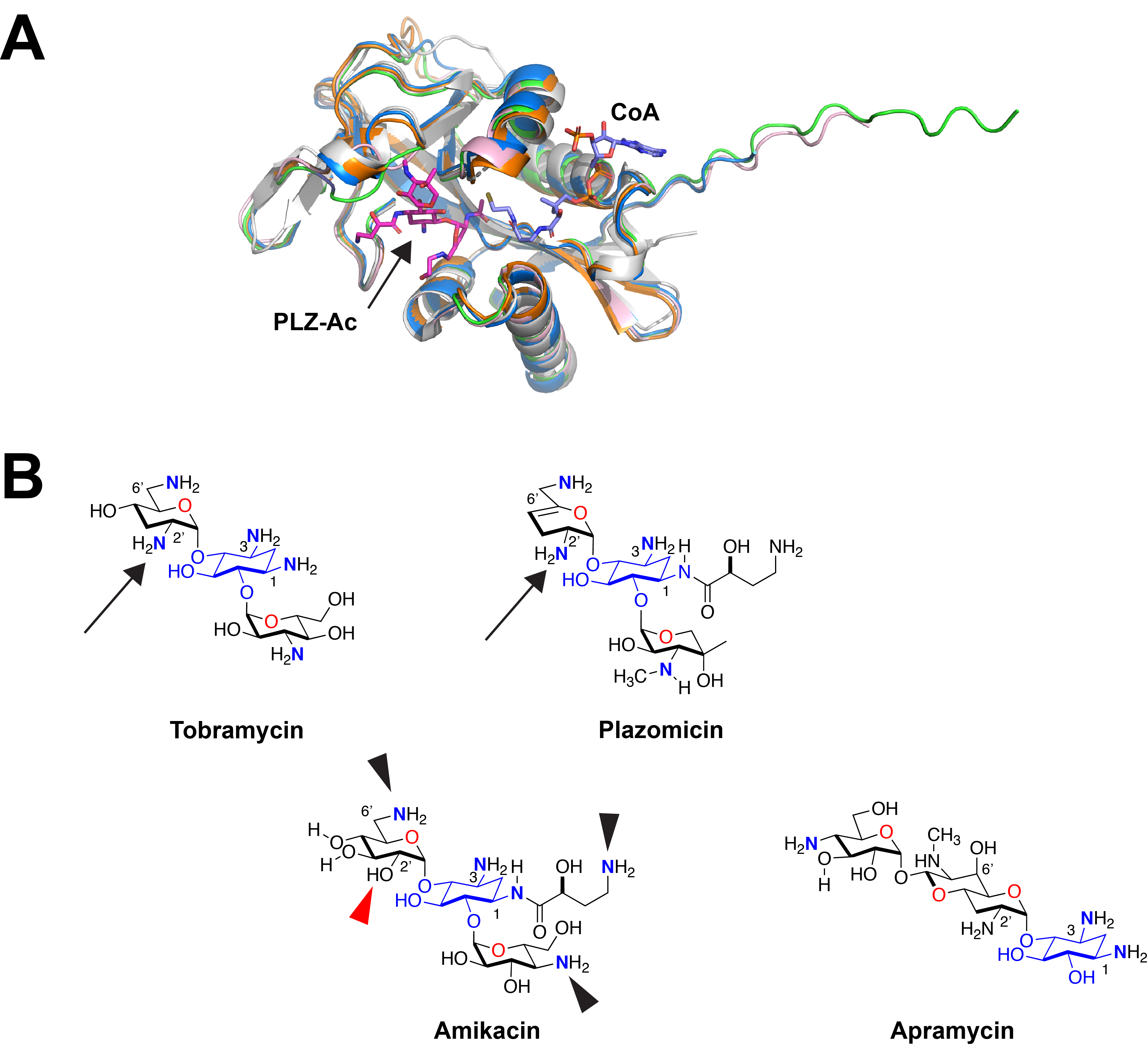
**

**Figure S1. Inactivation of aminoglycosides in *M. abscessus.* (A)** The structures of *M. abscessus* orf 4395 (a putative AAC(2') acetylase) and homologs from *M*. *fortuitum*, *M. chelonae*, and *M. tuberculosis* were predicted by ColabFold v1.5.5: AlphaFold2 using MMseqs2 ^1^. The highest ranked structures from each alpha-fold prediction were aligned with the *P. stuartii* aminoglycoside *N*-2'-acetyltransferase-Ia (AAC(2')-Ia) structure, PDB 6VOU ^2^. Shown are an overlay of aligned AAC(2')-Ia (gray) from the *P. stuartii* structure with acetylated plazomicin (PLZ-Ac, magenta) and coenzyme A (CoA, blue) with predicted protein homologues from *M. abscessus* ATCC 19977 (Mab Orf 4395, green); *M. fortuitum* DSM 46621 (orange); *M. chelonae* ATCC 35752 (light pink); and *M. tuberculosis* H37Rv (blue)*.* RMSD for all aligned pairwise comparisons with the *P. stuartii* enzyme were < 0.9 Å. Alignments and graphical representations were performed using Pymol version 3.1.3. **(B)** Chemical structures of apramycin, amikacin, plazomicin, and tobramycin rendered in ChemDraw version 22.2.0.3348. Positions in tobramycin and plazomicin presumptively modified by the AAC(2')-Ia homologue are indicated by arrows. Amikacin has a hydroxyl group in place of the primary amine at the *C*-2′ position (red dart), which eliminates this antibiotic as a substrate for AAC(2′)-Ia-mediated modification. The indicated primary amines in amikacin (black arrowheads) are identified sites of *N*-acetylation by the Eis enzyme from *M.* *tuberculosis* ^3^, which is highly homologous to the Eis2 enzyme in *M. abscessus* (alignment RMSD = 1.4 Å). Eis2 has been found responsible for the elevated amikacin MICs in the latter pathogen. ^4, 5^

**
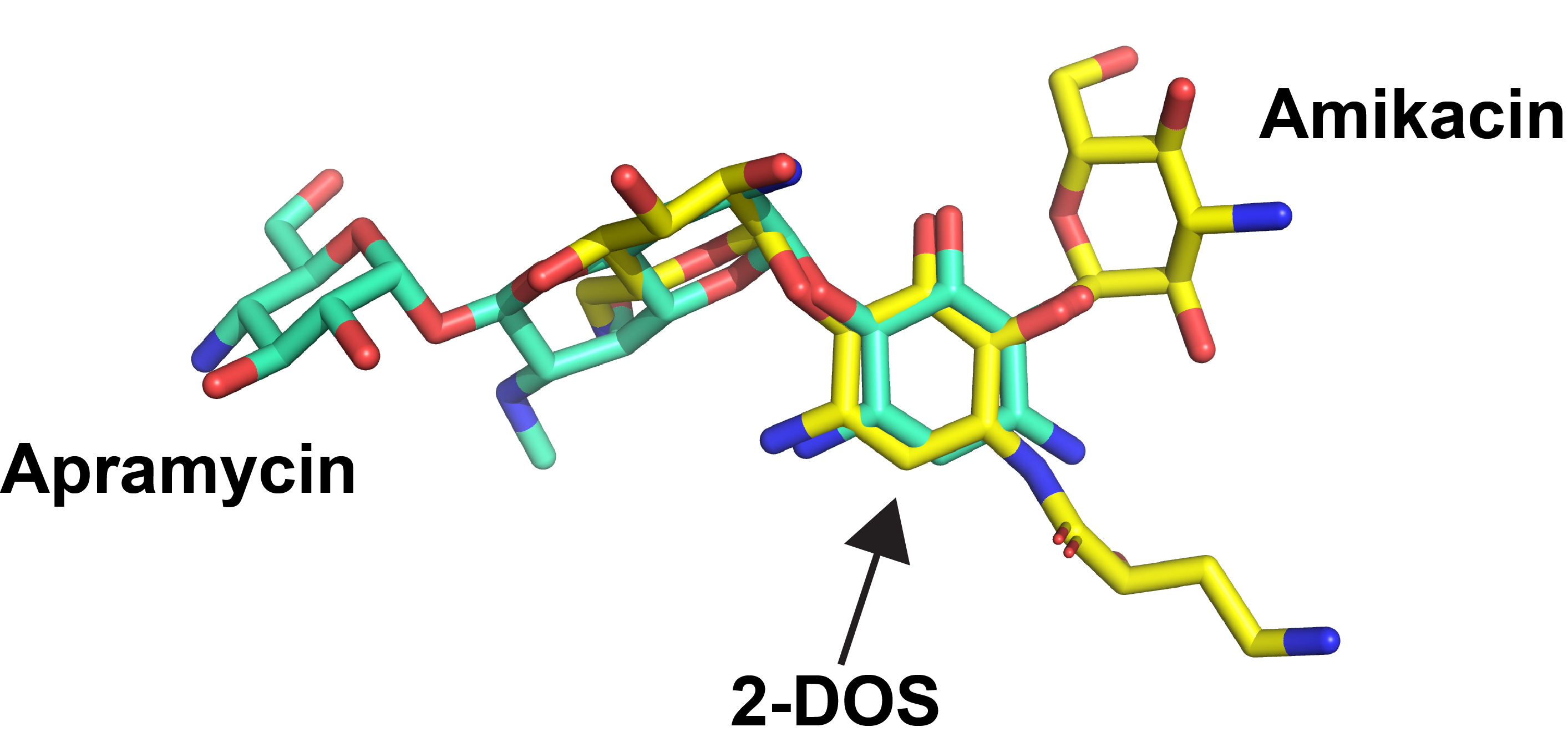
**

**Fig S2. Overlap of apramycin and amikacin binding sites in the prokaryotic ribosome.** Alignment and overlay of PDB 7PJS (apramycin, green) and 8SYL (amikacin, yellow). Both antibiotics bind to helix 44 of the 16S rRNA in the 30S ribosomal subunit. Their respective 2-deoxystreptamine (2-DOS) rings superimpose (arrow). Amikacin interacts with 16S rRNA G1405. In contrast, apramycin is shifted away from G1405, allowing it to make unique contacts with prokaryotic helix 44 in a region divergent from the corresponding mitochondrial ribosome structure, which may account for apramycin's more favorable side effect profile.

**Supplementary Figure References**

1. Mirdita M, Schütze K, Moriwaki Y *et al.* ColabFold: making protein folding accessible to all. *Nature Methods* 2022; **19**: 679-82.

2. Golkar T, Bassenden AV, Maiti K *et al.* Structural basis for plazomicin antibiotic action and resistance. *Commun Biol* 2021; **4**: 729.

3. Houghton JL, Biswas T, Chen W *et al.* Chemical and Structural Insights into the Regioversatility of the Aminoglycoside Acetyltransferase Eis. *ChemBioChem* 2013; **14**: 2127-35.

4. Selchow P, Ordway DJ, Verma D *et al.* Apramycin Overcomes the Inherent Lack of Antimicrobial Bactericidal Activity in Mycobacterium abscessus. *Antimicrob Agents Chemother* 2022; **66**: e0151021.

5. Ung KL, Alsarraf HMAB, Olieric V *et al.* Crystal structure of the aminoglycosides N-acetyltransferase Eis2 from Mycobacterium abscessus. *The FEBS Journal* 2019; **286**: 4342-55.

| **Table S1. *M. abscessus* isolate characteristics and MICs (mg/L)^c^ for apramycin, amikacin, plazomicin, tobramycin, clofazimine, and linezolid.** | | | | | | | | |
| --- | --- | --- | --- | --- | --- | --- | --- | --- |
| **Isolate No.** | **Subspecies^b^** | **Source** | **Apramycin** | **Amikacin** | **Plazomi-cin** | **Tobramy-cin** | **Clofazi-mine** | **Linezo-lid** |
| 11 | *abscessus* | bronchial washings | 16 | 32 | ND | 16 | 0.5 | 4 |
| 2 | *abscessus* | sputum | 4 | 16 | ND | 1 | 0.0625 | 2 |
| 3 | *abscessus* | sputum | 2 | 2 | ND | 16 | 0.25 | 1 |
| 7 | *abscessus* | bronchial washings | 1 | 2 | ND | 2 | 0.25 | 8 |
| 1 | *abscessus* | sputum/expectorated | 4 | 16 | ND | 8 | 0.25 | 2 |
| 8 | *massiliense* | sputum | 4 | 32 | ND | 8 | 0.25 | 32 |
| 5 | *massiliense* | sputum | 2 | 8 | ND | 8 | 0.125 | 1 |
| 18^a^ | *abscessus* | pleural fluid | 1 | 1 | ND | 4 | 0.125 | 1 |
| 23 | *abscessus* | tissue/hand | 4 | 16 | ND | 4 | 4 | 16 |
| 25 | *abscessus* | blood culture | 2 | 8 | ND | 2 | 0.5 | 8 |
| 26 | *abscessus* | sputum | 2 | 16 | ND | 1 | 0.125 | 4 |
| 27^a^ | *abscessus* | sputum | 2 | 8 | ND | 4 | 0.25 | 16 |
| 28^a^ | *abscessus* | bronchoalveolar lavage | 2 | 8 | ND | 8 | 4 | 16 |
| 29 | *abscessus* | sputum | 2 | 16 | ND | 8 | 8 | 8 |
| 31 | *abscessus* | olecranon bursa, elbow | 4 | 16 | ND | 8 | 0.5 | 32 |
| 33 | *abscessus* | sputum | 2 | 8 | ND | 8 | 0.25 | 16 |
| 34 | *abscessus* | sputum | 2 | 16 | ND | 8 | 0.25 | 4 |
| 35 | abscessus | tissue - manubrium | 2 | 16 | >16 | 2 | 0.125 | ND |
| 36 | *massilense* | sputum/induced | 2 | 16 | >16 | 32 | 0.5 | ND |
| 37 | *massilense* | sputum | 2 | 8 | >16 | 8 | 0.25 | 0.5 |
| 38 | *abscessus* | sputum/induced | 2 | 8 | >16 | 2 | 0.25 | ND |
| 39 | *abscessus* | sputum | 2 | 16 | >16 | 8 | 0.5 | 1 |
| 40 | *abscessus* | sputum/induced | 2 | 16 | >16 | 2 | 0.25 | 4 |
| 41 | *abscessus* | sputum | 2 | 16 | >16 | 16 | 8 | 1 |
| 42 | *abscessus* | bronchial washings | 2 | 16 | >16 | 4 | 0.25 | 2 |
| 43 | *abscessus* | bronchial washings | 4 | 16 | >16 | 8 | 0.25 | 2 |
| 44 | *abscessus* | bronchoalveolar lava | 2 | 16 | >16 | 8 | 0.25 | ND |
| 45 | *bollettii* | sputum | 2 | 16 | >16 | 8 | 1 | 0.5 |
| 47 | *massilense* | sputum | 2 | 0.5 | >16 | 8 | 1 | 1 |
| 48 | *abscessus* | bronchial washings | 2 | 16 | >16 | 4 | 2 | 1 |
| 50 | *abscessus* | sputum | 2 | 0.5 | >16 | 1 | 0.25 | ND |
| 51 | *massilense* | sputum | 4 | 16 | >16 | 16 | 2 | ND |
| 52 | *abscessus* | sputum | 4 | 16 | >16 | 8 | 0.25 | ND |
| 53 | *abscessus* | skin biopsy | 2 | 16 | >16 | 8 | 0.125 | 2 |
| 54 | *abscessus* | parotid gland | 4 | 16 | >16 | 8 | 0.25 | 16 |
| 55 | *abscessus* | bronchial washings | 4 | 16 | >16 | 8 | 0.25 | 1 |
| 56 | *abscessus* | sputum | 8 | 2 | >16 | 16 | 1 | 8 |
| 57 | *abscessus* | abdominal subcutaneous tissue | 2 | 0.25 | >16 | 4 | 0.25 | 0.5 |
| 58 | *abscessus* | abdominal wall | 4 | 16 | >16 | 8 | 0.25 | ND |
| 59 | *massilense* | sternal biopsy | 2 | 16 | >16 | 8 | 0.25 | 4 |
| 60 | *massilense* | sputum/induced | 4 | 16 | >16 | 8 | 8 | 4 |
| 62 | *abscessus* | sputum/induced | 4 | 16 | >16 | 8 | 0.25 | 16 |
| 63 | *abscessus* | left lower quandrant abdominal collection | 2 | 16 | >16 | 8 | 4 | 4 |
| 66 | *massilense* | bronchial washings | 2 | 32 | >16 | 8 | 4 | 1 |
| 67 | *abscessus* | sputum | 2 | 32 | >16 | 4 | 0.5 | ND |
| 68 | *massilense* | bronchial washings | 4 | 16 | >16 | 8 | 2 | 2 |
| 69 | *massilense* | sputum/expectorated | 16 | 2 | >16 | 4 | 8 | ND |
| 70 | *abscessus* | bronchial washings | 2 | 32 | >16 | 2 | 0.25 | 1 |
| 71 | *abscessus* | sputum/induced | 2 | 0.0625 | >16 | 4 | 0.5 | 1 |
| 72 | *massilense* | sputum | 2 | 8 | >16 | 2 | 2 | 1 |
| 73 | *massilense* | sputum | 2 | 16 | >16 | 4 | 0.25 | ND |
| 74 | *massilense* | right upper lobe lung nodule | 2 | 16 | >16 | 8 | 0.25 | ND |
| 75 | *abscessus* | sputum | 4 | 16 | >16 | 16 | 8 | 16 |
| 76 | *abscessus* | sputum/induced | 4 | 4 | >16 | 16 | 4 | 1 |
| 77 | *abscessus* | bronchial washings | 2 | 8 | >16 | 4 | 4 | 1 |
| 78 | *massilense* | bronchial washings | 4 | 16 | >16 | 8 | 0.5 | ND |
| 80 | *abscessus* | sternal soft tissue swab | 2 | 16 | >16 | 4 | 0.25 | ND |
|  |  | n | 57 | 57 | 40 | 57 | 57 | 43 |
|  |  | median | 2 | 16 | >16 | 8 | 0.25 | 2 |
|  |  | MIC90 | 2 | 16 | >16 | 16 | 4 | 32 |
|  |  | range low | 1 | 1 | >16 | 1 | 0.0625 | 1 |
|  |  | range high | 16 | 32 | >16 | 32 | 8 | 32 |
|  |  | percent presumptively susceptible^d^ | **96.5%** | **91.2%** | **0.0%** | **17.5%** |  | **79.1%** |
|  |  |  |  |  |  |  |  |  |
|  |  |  |  |  |  |  |  |  |

^a^Isolates used in checkerboard studies summarized in Table 2. Isolates 27 and 28 were used in time-kill studies shown in Figs. 1 and 2 and spontaneous resistance studies shown in Table 3.

^b^Subspecies determined by the method of Akwani WC, van Vliet AHM, Joel JO, Andres S, Diricks M, Maurer FP, et al. The Use of Comparative Genomic Analysis for the Development of Subspecies-Specific PCR Assays for *Mycobacterium abscessus*. Front Cell Infect Microbiol. 2022;12:816615. https://doi.org/10.3389/fcimb.2022.816615.

^c^MIC values shown are the modal value of three biological replicates.

^d^Amikacin breakpoints MIC ≤ 16 S, 32 I, ≥64 R; tobramycin breakpoints MIC ≤ 2 S, 4 I, ≥8 R; linezolid breakpoints ≤8 S, 16 I, ≥32 R per CLSI M62. Proposed apramycin susceptibility breakpoint of ≤ 8 mg/L based on extrapolation of 99% probability of 2log10 CFU reduction for carbapenem-resistant *A. baumannii* in a murine pneumonia model^1^ modified by the modestly lower than predicted BAL epithelial lining fluid AUC/infinity measurements in a human phase I clinical trial, NCT05590728, at a 30 mg/kg single dose.^2^ Results are highlighted as green, susceptible; yellow, intermediate or non-susceptible; red, resistant for these available breakpoints. Although amikacin MIC ≤ 16 mg/L is considered susceptible for rapidly growing mycobacterium per 2018 CLSI M62, it should be noted that revised amikacin breakpoints for Enterobacterales per 2024 CLSI M100 are ≤4 mg/L with caveats.

| **Table S2. *M. chelanae* and *M. fortiuitum* MICs (mg/L)^b^ for apramycin, amikacin, and tobramycin.** | | | | | |
| --- | --- | --- | --- | --- | --- |
| **Species** | **Isolate #** | **Source** | **Apramycin** | **Amikacin** | **Tobramycin** |
| *M. chelonae* | 13 | sputum | 2 | 16 | 8 |
| *M. chelonae* | 14 | sputum | 1 | 8 | 1 |
| *M. chelonae* | 16A^a^ | fluid, other/abdomen | 1 | 8 | 8 |
| *M. chelonae* | 16B^a^ | fluid, other/abdomen | 1 | 8 | 8 |
| *M. fortuitum* | 4B | sputum | 8 | 8 | 8 |
| *M. fortuitum* | 9A | sputum | 4 | 1 | 8 |
| *M. fortuitum* | 9B | sputum | 2 | 2 | 16 |
| *M. fortuitum* | 10 | sputum | 2 | 16 | 1 |
| *M. fortuitum* | 12 | sputum | 1 | 2 | 8 |
| *M. fortuitum* | 17A | sputum | 2 | 8 | 4 |
| *M. fortuitum* | 17B | sputum | 0.5 | 2 | 1 |
| *M. fortuitum* | 20A | sputum | 2 | ≤0.5 | 4 |
| *M. fortuitum* | 20B | sputum | 1 | 2 | 8 |
| *M. fortuitum* | 30 | sputum | 4 | 2 | 16 |
| *M. fortuitum* | 32A | sputum | 0.5 | 0.5 | 0.0625 |
| *M. fortuitum* | 32B | sputum | 2 | 2 | 8 |
|  |  | n | 16 | 16 | 16 |
|  |  | MIC50 | 2 | 2 | 8 |
|  |  | MIC90 | 4 | 16 | 16 |
|  |  | range low | 0.5 | 0.5 | 0.0625 |
|  |  | range high | 8 | 16 | 16 |
|  |  | percent presumptively susceptible^c^ | **100.0%** | **100.0%** | **25.0%** |

^a^Isolates with the same numerical designation but with different suffix (A versus B) were isolated from the same specimen, but were tested separately based on distinct colony morphologies.

^b^MIC values shown are the modal value of three biological replicates.

^c^Amikacin breakpoints MIC ≤ 16 S, 32 I, ≥64 R; tobramycin breakpoints MIC ≤ 2 S, 4 I, ≥8 R; linezolid breakpoints ≤8 S, 16 I, ≥32 R per CLSI M62. Proposed apramycin susceptibility breakpoint of ≤ 8 mg/L based on extrapolation of 99% probability of 2log10 CFU reduction for carbapenem-resistant *A. baumannii* in a murine pneumonia model^1^ modified by the modestly lower than predicted BAL epithelial lining fluid AUC/infinity measurements in a human phase I clinical trial, NCT05590728, at a 30 mg/kg single dose.^2^ Results are highlighted as green, susceptible; yellow, intermediate or non-susceptible; red, resistant for these available breakpoints. Although amikacin MIC ≤ 16 mg/L is considered susceptible for rapidly growing mycobacterium per 2018 CLSI M62, it should be noted that revised amikacin breakpoints for *Enterobacterales* per 2024 CLSI M100 are ≤4 mg/L with caveats.

**Supplementary Table References**

1. Becker K, Aranzana-Climent V, Cao S, Nilsson A, Shariatgorji R, Haldimann K, Platzack B, Hughes D, Andrén PE, Böttger EC, Friberg LE, Hobbie SN; ENABLE consortium. Efficacy of EBL-1003 (apramycin) against *Acinetobacter baumannii* lung infections in mice. Clin Microbiol Infect. 2021 Sep;27(9):1315-1321. doi: 10.1016/j.cmi.2020.12.004. Epub 2020 Dec 11. PMID: 33316399.

2. Anonymous. Safety of Intravenous Apramycin in Adults, NCT05590728, on ClinicalTrials.gov. https://clinicaltrials.gov/study/NCT05590728?term=apramycin&rank=1&tab=results. Accessed April 8, 2025.
